# Heterogeneous and Photosensitized Oxidative Degradation Kinetics of the Plastic Additive Bisphenol-A in Sea Spray Aerosol Mimics

**DOI:** 10.1021/acs.jpca.3c00127

**Published:** 2023-05-18

**Authors:** Samantha M. Kruse, Jonathan H. Slade

**Affiliations:** Department of Chemistry and Biochemistry, University of California, San Diego, La Jolla, California 92093, United States

## Abstract

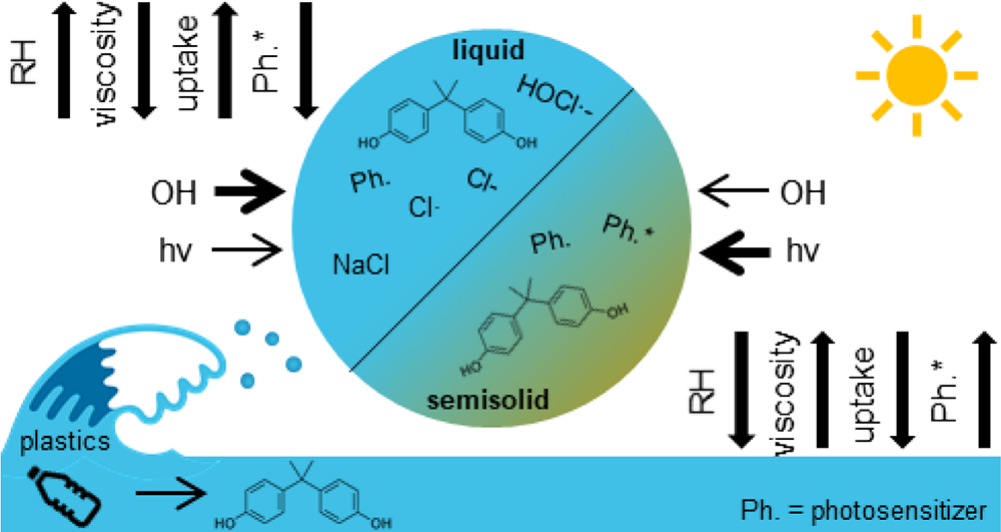

Plastics have become ubiquitous in the world’s oceans, and recent work indicates that they can transfer from the ocean to the atmosphere in sea spray aerosol (SSA). Hazardous chemical residues in plastics, including bisphenol-A (BPA), represent a sizable fraction of consumer plastics and have been measured consistently in air over both terrestrial and marine environments. However, the chemical lifetimes of BPA and mechanisms by which plastic residues degrade with respect to photochemical and heterogeneous oxidation processes in aerosols are unknown. Here, we present the photosensitized and OH-initiated heterogeneous oxidation kinetics of BPA in the aerosol phase consisting of pure-component BPA and internal mixtures of BPA, NaCl, and dissolved photosensitizing organic matter. We found that photosensitizers enhanced BPA degradation in binary-component BPA + photosensitizer aerosol mixtures when irradiated in the absence of OH. OH-initiated degradation of BPA was enhanced in the presence of NaCl with and without photosensitizing species. We attribute this enhanced degradation to greater mobility and thus reaction probability between BPA, OH, and reactive chlorine species (RCS) formed through reaction between OH and dissolved Cl^–^ in the more liquid-like aerosol matrix in the presence of NaCl. Addition of the photosensitizers in the ternary-component BPA + NaCl + photosensitizer aerosol led to no enhancement in the degradation of BPA following light exposure compared to the binary-component BPA + NaCl aerosol. This was attributed to quenching of triplet state formation by dissolved Cl^–^ in the less viscous aqueous aerosol mixtures containing NaCl. Based upon measured second-order heterogeneous reaction rates, the estimated lifetime of BPA with respect to heterogeneous oxidation by OH is one week in the presence of NaCl compared to 20 days in the absence of NaCl. This work highlights the important heterogeneous and photosensitized reactions and the role of phase state, which affect the lifetimes of hazardous plastic pollutants in SSA with implications for understanding pollutant transport and exposure risks in coastal marine environments.

## Introduction

An estimated 5% of the 275 million metric tons (MT) of annually produced plastics, 4.8 to 12.7 MT, accumulate in the world’s oceans.^1^ Epoxy resins and hardeners in plastics, including bisphenols such as bisphenol-A (BPA), represent 10 to 70% of the mass of additives in plastics and have known carcinogenic and teratogenic properties.^2,3^ BPA can leach from plastics into the surrounding water and, due to its hydrophobicity and lipophilicity, accumulate at the ocean surface along with other organic material and inorganic salts. BPA concentrations in ocean water have been measured in the range of 0.04–17,000 μg/L depending on proximity to terrestrial inputs and are found to be more persistent in ocean water than in freshwater.^4−6^ BPA is ubiquitous in the atmosphere with mass concentrations in the range of 1–17,400 pg m^–3^ in atmospheric aerosol sampled across urban, rural, marine, and polar regions.^7^ A major source of atmospheric BPA is through plastic incineration, although a non-negligible portion (1–32 pg m^–3^) has been measured in aerosol over the remote ocean.^7^

Non-volatile contaminants at the ocean surface can become airborne via selective transfer in sea spray aerosol (SSA), which is composed of a complex mixture of inorganic salts, fatty acids, saccharides, and chromophoric (light-absorbing) dissolved organic matter that accumulate at the sea surface microlayer.^8−11^ SSA contributes the largest aerosol flux by mass to the atmosphere per year. In recent work, SSA was found to be an important source of airborne plastics, with an estimated emission flux from the ocean of up to 22 megatons annually.^12^ Due to its relatively high lipophilicity (logP = 3.3), BPA is expected to selectively transfer into SSA following the rupture of films produced by bubbles at the ocean surface, which are enriched in surfactants and other lipophilic material.^13,14^ Currently, the chemical lifetimes and pathways by which contaminant molecules such as BPA degrade in the presence of SSA constituents are unknown.

In the atmosphere, aerosols undergo chemical aging through photochemical and heterogeneous reaction pathways when exposed to sunlight and atmospheric oxidants including ozone, OH radicals, and Cl radicals.^15−17^ Oxidation reactions can degrade organic components in aerosol and alter aerosol particle physicochemical properties, such as hygroscopicity, leading to greater wet depositional losses.^18−21^ In addition to heterogeneous oxidation reactions, photosensitized reactions in atmospheric aerosols can promote further oxidative degradation via formation of transient reactive oxygen species^22,23^ or by enhancing the reactive uptake of atmospheric oxidants including ozone.^24^ These reactions that alter the chemical composition of the aerosol can also modulate their toxicity, having implications for public health.^25^ Photosensitizers play an important role in the marine-atmospheric environment and at the sea surface due to their accumulation at the sea surface and ability to transfer into SSA.^9,26,27^ Prior work has shown that BPA can undergo enhanced degradation in the presence of photosensitizers in bulk water.^28^ However, the photochemical and oxidative degradation pathways, reactive uptake kinetics by OH, and degradation kinetics of plastic additives including BPA in marine-relevant aerosol matrices have not been studied.

In this work, we examine the photochemical and heterogeneous oxidative degradation pathways and kinetics of BPA following OH reactive uptake to model SSA mixed with BPA. Lab-generated SSA “mimics” consisting of BPA, NaCl, and different photosensitizing organic materials, including 4-benzoylbenzoic acid (4-BBA) and humic acid (HA), were irradiated in an oxidation flow reactor at varying exposure levels of light and OH. An extractive electrospray ionization time-of-flight mass spectrometer (EESI-TOF) was employed to measure aerosol composition and the decay of BPA in real time following exposure to light and OH. This work highlights the importance of NaCl and photosensitizers as reactive species in the degradation of emerging contaminant molecules present in plastics that become airborne over the ocean with important implications regarding the persistence of organic contaminants in SSA.

## Methods

An extractive electrospray ionization time-of-flight mass spectrometer (EESI-TOF; Aerodyne Research Inc. and Tofwerk AG) was utilized to measure the concentrations of BPA and its oxidation products in the aerosol phase. EESI-TOF was used in our previous analysis of SSA organic components and in other studies for the analysis of secondary organic aerosols.^18,29−32^ Briefly, a reagent ion spray was generated by passing the reagent solution through a 365 μm OD fused silica capillary (IDEX Health and Science, LLC) at a pressure of 350 mbar and charged at a voltage of −2500 V. The EESI-TOF was operated in negative mode using a reagent solution of 2% (by mass) acetic acid (99.7%, Fisher Chemical), 48% H_2_O (18 mΩ, Millipore Synergy System), and 50% acetonitrile (≥99.95%, Fisher Chemical) spiked with 100 ppm sodium iodide (99%, Sigma-Aldrich) for the purposes of mass calibration. The aerosol was sampled through the EESI-TOF inlet at a rate of 1 liter per minute (LPM) and mixed orthogonally with the reagent ion spray. The mixed aerosol/reagent ion droplets were heated and volatilized at a temperature of 220 °C.^29^ Under these conditions, ionization occurred primarily via deprotonation as C_15_H_15_O_2_^–^.

Solutions of 25/75 (% by mass) water/methanol (≥99.9%, Fisher Chemical) were prepared with 100 ppm BPA (≥99%, Sigma-Aldrich), 4-benzoylbenzoic acid (4-BBA; 99%, ACROS Organics), humic acid (HA; ≥95%, Alfa Aesar), and sodium chloride (99.5%, Fisher Chemical). Under these conditions, the pH of the atomizer solution was neutral for pure-component BPA and BPA + NaCl solutions and slightly more acidic when mixed with 4-BBA and HA. It is assumed because the p*K*_a_ = 10.6 for BPA that BPA remained in its molecular form for all experiments. However, the predicted p*K*_a_ = 3.79 of 4-BBA indicates that it would have been present in its carboxylate form. Particle- and hydrocarbon-free zero air (Airgas) was used as a carrier flow. The solution was then atomized by zero air using a constant output atomizer (Model 3076, TSI Inc.) and sent through a silica bead diffusion dryer and an activated charcoal denuder to dry the aerosol particles and remove volatile species. The aerosol was then neutralized and size selected by a differential mobility analyzer (Model 2100; Brechtel) to generate monodisperse aerosol with a nominal electrical mobility diameter of 100 nm to maintain size consistency across experiments.

The aerosol was exposed to differing levels of light, ozone, and OH in a potential aerosol mass oxidative flow reactor (PAM-OFR; Aerodyne Research Inc.).^33−36^ The PAM-OFR was equipped with four interchangeable, narrowband lamps either operated at wavelengths of 254 nm (GPH436T5L; Light Sources, Inc.) or 369 nm (F436T5/BLC/4P-369; Aerodyne Research, Inc.) and arranged concentrically along the inner walls and protected by a quartz sheath tube and cooled with a gentle flow of nitrogen. The light intensity inside the PAM-OFR was adjusted with a ballast and measured with a photodetector (TOCON-C6, sglux GmbH). OH was generated via the photolysis of ozone in the presence of water vapor and 254 nm radiation in a mix with zero air (RH ∼ 80% and air flow rate of 3 LPM). Ozone was generated in an isolated external chamber via photolysis of O_2_ in a flow of zero air (1 LPM) in the presence of 185 nm radiation. Ozone concentrations were measured with an ozone monitor (106-M, 2B Technologies) and were 1.2 (±0.1) ppm for all experiments. OH concentrations in the PAM-OFR were calibrated by measuring the decay of CO with a CO monitor (Model APMA-370, Horiba) at the different lamp voltage settings and were varied from 1.5 to 5.0 × 10^9^ molecules cm^–3^. This concentration range of OH is 2–3 orders of magnitude greater than ambient OH levels but chosen to achieve the equivalent of up to a week of aging in the atmosphere at the residence time in the PAM-OFR of 123 s. At higher concentrations of OH, the rate of OH uptake by the aerosol may be affected, although other studies have shown that the elemental composition of aerosol is similar whether at low concentrations of OH over long exposure times or high concentrations of OH over short exposure times.^37,38^

Losses of BPA resulting from exposure to ozone were insignificant and no further corrections were applied. While heterogeneous OH oxidation was conducted in the presence of 254 nm radiation, separate control experiments were conducted to determine the photodegradation rates of BPA by 254 nm light in the absence of OH. The additional losses of BPA resulting from photodegradation in the presence of 254 nm light alone were subtracted from the losses of BPA in the presence of OH. Studies of the photosensitized degradation kinetics of BPA were conducted in the presence of 369 nm light in the absence of OH and 254 nm light, representing more relevant conditions of irradiation in the troposphere.

All mass spectrometer datasets were processed in Tofware version 3.2.2 (Aerodyne LLC and Tofwerk AG), which ran in Igor Pro 8 (Wavemetrics). Mass spectral peaks were assigned based upon elemental formulae applying high-resolution peak fitting analysis at a nominal mass resolution (*m/*Δ*m*) ∼3000 for *m/z* 59 (CH_3_COO^–^) in negative mode. Signal intensities were corrected for drift applying a linear interpolation of the ion intensity of BPA measured prior to and after exposure to OH or light. Potential changes in sensitivity were accounted for by introducing an “internal standard” of BPA aerosol directly to the EESI-TOF inlet (bypassing the PAM-OFR) between different OH exposure levels. The intensity of the BPA signal measured at each OH exposure level was then normalized to the ratio of the internal standard signal measured before and after each OH exposure level, as shown in Figure S1 and described in more detail in the SI. Background signal intensities (blanks) were acquired at the start and end of each trial after passing the aerosol flow through a HEPA filter (99.99% removal efficiency at 0.1 μm) and were subtracted from the sample signal intensities of BPA.

Heterogeneous reactive uptake kinetics were assessed by measuring the diffusion-corrected reactive uptake coefficient (γ), defined here as the ratio of reactive collisions to total collisions between OH and the aerosol that result in the loss of BPA.^37,39,40^ γ has been determined previously to evaluate such heterogeneous processes as the uptake kinetics of water onto acidic aqueous surfaces and the uptake of trace reactive gases by various organic aerosol compounds.^15,37,39,41,42^ γ was calculated using eq 1:
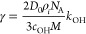
1Here, *D*_0_ is the mean surface area-weighted aerosol diameter, ρ_*i*_ is the density of BPA, *N*_A_ is Avogadro’s number, *c*_OH_ is the average speed of OH in the gas phase, and *M* is the molecular weight of BPA. *k*_OH_ is the measured second-order heterogeneous loss rate, determined via the slope of the natural logarithm of the normalized BPA signal as a function of the OH exposure level measured in the PAM-OFR. γ was corrected for OH diffusion losses following the approach by Sutugin and Fuchs,^43^ resulting in less than 1% correction at 1 atm.

## Results and Discussion

### Pure-Component BPA Degradation by OH

**Figure 1:**
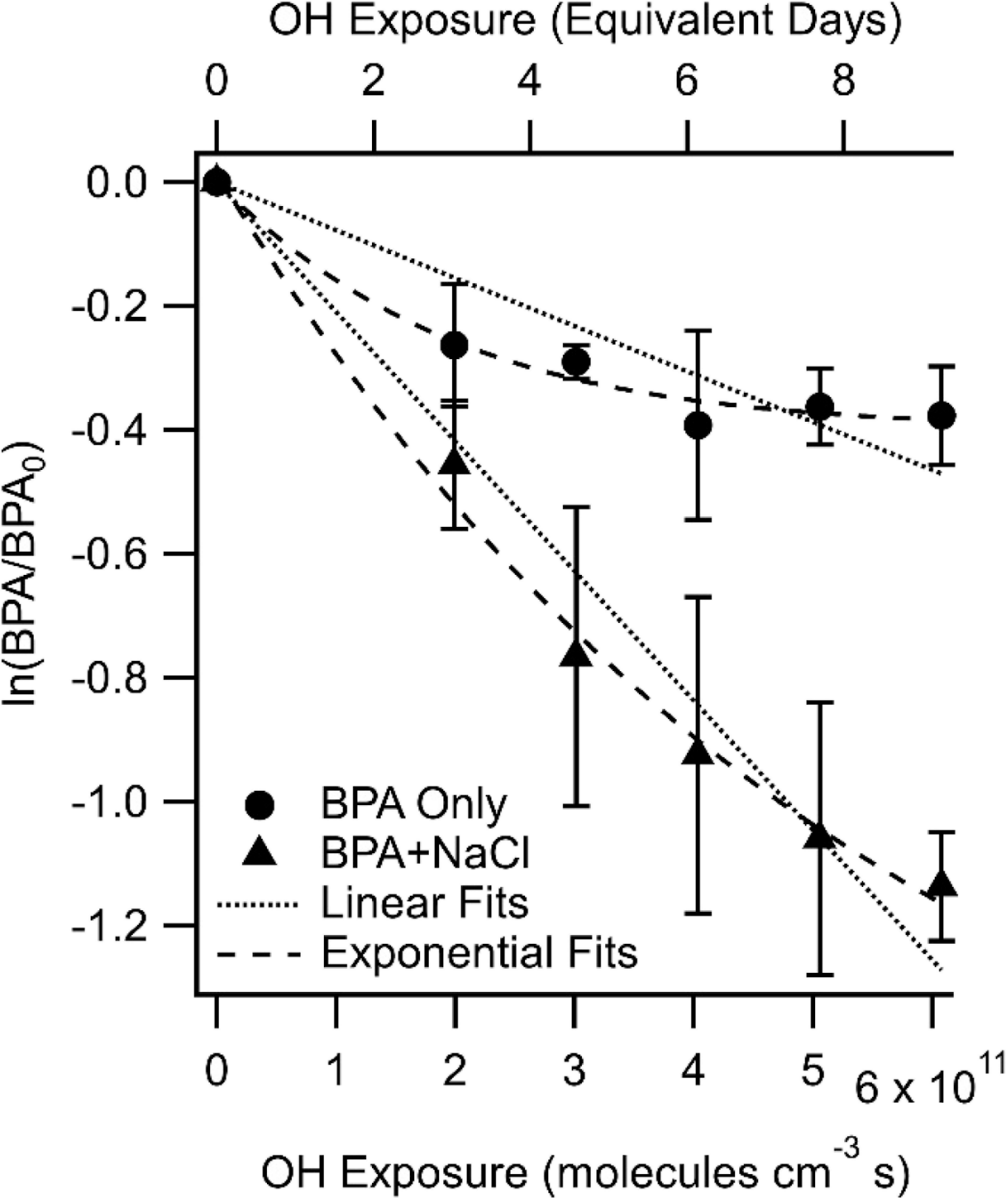
Natural logarithm of the fractional decay of BPA as a function of OH exposure averaged over multiple experiments for BPA (circles) and BPA + NaCl (triangles). The slope of the exponential fit was a parameterization from Davies and Wilson et al. (large dashed line).^44^ The slope of the linear fit (small dashed line) of the decay up to 6.1 × 10^11^ molecules s cm^–3^ of OH exposure represents the second-order heterogeneous reaction rate constant for reactive OH uptake by BPA aerosol. The top axis corresponds to the equivalent amount of aging in days assuming an ambient steady-state OH concentration of 1.5 × 10^6^ molecules cm^–3^.

Pure-component BPA aerosol was exposed to different OH exposure levels ranging from 0 to 6.1 × 10^11^ molecules s cm^–3^ corresponding to the equivalent of 0 to 9.4 days of aging in the atmosphere assuming an average daily OH concentration of 1.5 × 10^6^ molecules cm^–3^ (Figure 1). The heterogeneous oxidation kinetics of BPA were determined by plotting the natural logarithm of the normalized BPA signal (*m*/*z* = 227.1077533 Th) as a function of OH exposure, as shown in Figure 1. The slope of the fit to OH exposure is the effective second-order heterogeneous reaction rate constant (*k*_OH_) expressed in cm^3^ molecule^–1^ s^–1^, which is reported in Table 1 for all experiments.^39^ As noted in the Methods, some of the BPA loss in the aerosol phase is attributed to direct photolysis by exposure to the 254 nm lamps in the PAM-OFR.^45^ This loss, which was in addition to the reactive losses by OH, was subtracted from the measured decay when BPA was exposed to OH and 254 nm radiation. Following this correction and applying the linear fit, as shown in Figure 1, the effective *k*_OH_ = 0.773 (±0.082) × 10^–12^ cm^3^ molecule^–1^ s^–1^. This result is comparable with previous studies of OH heterogeneous oxidation of different organic aerosol compounds, which are on the order of 1.0 × 10^–12^ cm^3^ molecule^–1^ s^–1^.^37^

**Table 1:** Experimentally-Derived *k*_OH_, *k*_254nm_, *k*_369nm_, γ, and Atmospheric Lifetime in Days

experiment	*k*_OH_ (10^–12^ cm^3^ molecules^–1^ s^–1^)	*k*_254nm_ (10^–3^ s^–1^)	*k*_369nm_ (10^–3^ s^–1^)	γ	BPA lifetime by OH reaction (days)
BPA	0.773 ± 0.082	1.153 ± 0.398	1.457 ± 1.027	0.211 ± 0.022	20.0
BPA + NaCl	2.091 ± 0.098	1.928 ± 1.626	1.711 ± 1.418	0.398 ± 0.019	7.38
BPA + NaCl + 4-BBA	2.016 ± 0.064	2.105 ± 0.772	0.545 ± 0.512	0.546 ± 0.017	7.66
BPA + NaCl + HA	2.062 ± 0.069	1.801 ± 0.586	1.168 ± 0.843	0.580 ± 0.019	7.48
BPA + 4-BBA	1.226 ± 0.047	5.381 ± 1.797	7.050 ± 3.821	0.313 ± 0.012	12.6
BPA + HA	0.791 ± 0.008	5.577 ± 1.373	3.478 ± 1.375	0.210 ± 0.002	19.5

Our calculated γ = 0.211 (±0.002) is of the same magnitude but lower when compared to other reported γ values for OH uptake by single-component and mixed organic aerosol. In the case of water-soluble organic aerosols such as levoglucosan, γ = 0.65 (±0.17) at 40% RH and γ = 0.21 (±0.18) at 0% RH, while OH uptake by erythritol was measured to be γ = 0.77 (±0.10).^19,37^ In contrast, sparingly soluble organic aerosol components including 4-methyl-5-nitrocatechol, exhibited slower OH uptake kinetics with γ = 0.07 (±0.02) at RH = 26%.^19^ The reactive uptake kinetics of OH have also been shown to depend on the concentration of OH via competitive reactions between co-adsorbed species, including OH with itself and reactions between OH and co-adsorbed O_3_ or intermediate products, which occupy reactive sites for OH at the surface.^37,46,47^ In our experiments, it is likely that the relatively high [O_3_] ∼1 ppm affected the reactive uptake kinetics of OH but not unlike in other studies of heterogeneous oxidation kinetics using the PAM-OFR, which employed similarly large [O_3_] and [OH].^33^ Furthermore, [O_3_] and [OH] were the same for all experiments, and therefore it would not cause the relatively slower kinetics between OH and BPA compared to the other aerosol mixtures studied here. While the experiments presented here were performed at RH = 80%, the very low water solubility of BPA suggests that the particles remained in a highly viscous or solid-like phase state, which conceivably could have slowed the rates of molecular diffusion and thus the extent of reaction between OH and BPA in the particle phase. Evidence of this is demonstrated in Figure 1 for pure-component BPA, which did not significantly further degrade in the aerosol phase above an OH exposure level of ∼4 × 10^11^ molecules s cm^–3^, whereas the aqueous particle mixture with NaCl led to further degradation of BPA. In the work by Davies and Wilson, reactive uptake of OH by citric acid decreased with increasing OH exposure level when RH < 50%, whereas the reactive uptake of OH was enhanced when the RH was greater than 50%, attributed to better mixing of reactive components in the less viscous aqueous aerosol phase.^44^ Based upon the applied parameterized fits in Figure 1 using the unreacted core-reactive shell model by Davies and Wilson, even at the high RH, apparent mixing lengths of BPA in the pure-component BPA aerosol were not considered well mixed. Further descriptions of this parameterization are provided in the SI but what this analysis indicated was that unreacted BPA was not readily replenished at the particle surface for reaction with OH.

**Figure 2:**
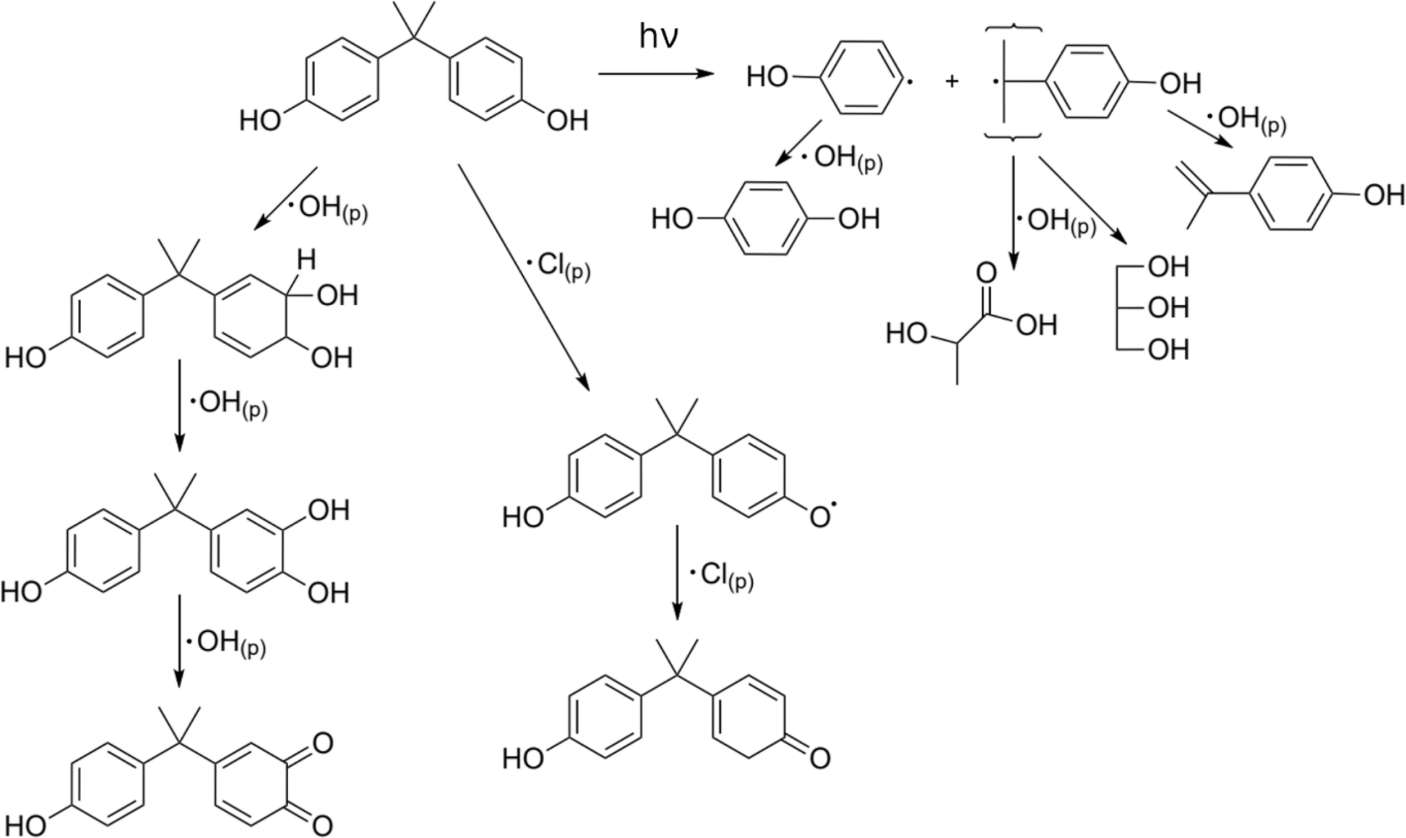
Dominant pathways of BPA degradation via OH and Cl radicals and light.^28,48,49^

The oxidative degradation of BPA by OH is expected to proceed via addition to the phenol ring generating a catechol.^49^ Exposure to 254 nm radiation is known to decompose BPA by breaking the carbon–carbon bond between the quaternary carbon and carbon on the phenol group, and subsequent oxidation of these fragmented products by OH can form smaller molecular weight oxidation products.^28^ These mechanisms are summarized in Figure 2. These products were not detected in the mass spectral data above the experimental background, which could result from the degradation of the first-generation oxidation products at the relatively high OH exposure levels in the PAM-OFR that subsequently fragment and volatilize from the aerosol surface and therefore would not be detected in the aerosol phase by the EESI-TOF. As shown in Figure S2, ∼3% of the particle mass was lost due to volatilization at the equivalent of 9.4 days of OH exposure. This indicates a shift in reaction pathways of BPA degradation by OH from functionalization at the lowest OH exposures (up to 3 × 10^11^ molecules s cm^–3^) to fragmentation as the OH exposure was increased from 3 × 10^11^ to 6 × 10^11^ molecules s cm^–3^.

### Degradation of BPA in the Presence of NaCl by OH and the Role of Phase State

**Figure 3:**
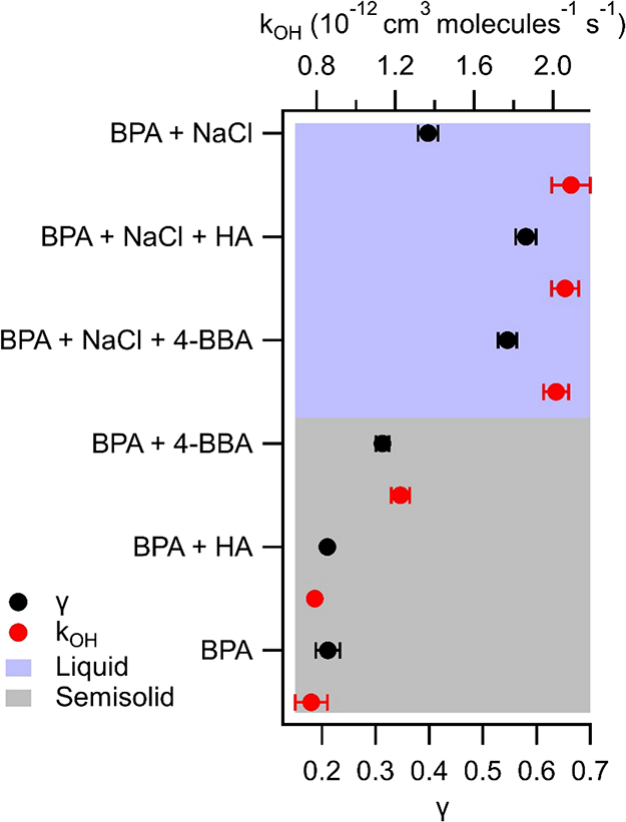
Experimentally derived second-order rate constants of BPA degradation in aerosol by OH reactive uptake (*k*_OH_; red) and the corresponding OH reactive uptake coefficients (γ; black) for all aerosol systems studied. Error bars represent the standard deviations between multiple experiments. The blue shading represents experiments where the aerosols were calculated to be liquid (viscosity < 10^2^ Pa s), and the gray shading represents experiments where they were calculated to be semisolid (viscosity > 10^2^ Pa s).

Sea salt (NaCl) was mixed with BPA (50:50 by wt %) in the atomizer solution to mimic the bulk chemical composition of SSA. In the presence of NaCl, heterogeneous loss of BPA by reactive uptake of OH increased significantly in comparison to the pure-component BPA aerosol. The measured *k*_OH_ = 2.091 (±0.098) × 10^–12^ cm^3^ molecule^–1^ s^–1^, and γ = 0.398 (±0.019), a factor of 1.8 greater reactive loss of BPA when in the presence of NaCl, as shown in Figure 3. Previous work has also observed increased BPA degradation in aqueous systems containing NaCl, in which it is suggested that this is due to the generation of HOCl·^–^ and other reactive species that would otherwise not form in the absence of NaCl.^50^ This is the first such observation of enhanced heterogeneous reactive loss of an organic component in the aerosol phase upon exposure to OH in the presence of NaCl. In the work of Trueblood et al., heterogeneous OH oxidative aging of real SSA in a PAM-OFR led to a significant depletion in the fraction of the non-volatile organic components in the aerosol phase.^20^ This was attributed to direct OH oxidation of the organic species in sea spray including amino acids that resulted from C–C bond scission. The effects of NaCl were not accounted for in that study. In the work of Sakamoto et al., which measured the uptake of OH onto pure deliquesced NaCl aerosol, γ varied from 0.77 to 0.95, which is within the reported range of γ in this study for the aerosol mixtures containing NaCl measured above the deliquescence relative humidity (DRH ∼ 75%) of NaCl.^51^ They attribute the greater uptake to greater diffusion and reaction in the aerosol bulk in the deliquesced state.

In the presence of NaCl, the degradation of BPA in the aerosol phase following OH uptake may be further impacted by the formation of reactive chlorine species (RCS), i.e., Cl radicals, Cl radical-ion species, and chlorine gas, as shown in eqs 2–5, which subsequently could have reacted with neighboring BPA molecules in the aerosol phase, as shown in Figure 2.^52^
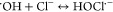
2
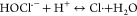
3
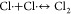
4
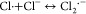
5

As shown in Figure S3, we observed depletion of the Cl^–^ signal (*m*/*z* = 34.969401) in the EESI-TOF following exposure to OH when NaCl was present in the aerosol phase. This depletion of Cl^–^ is indicative of heterogeneous reactions between OH and Cl^–^ and thus formation of RCS in the aerosol phase. Like the reaction with OH, the Cl radical is expected to abstract a hydrogen atom from the −OH on the phenol or of BPA. Lei et al. demonstrated for several phenolic compounds, including BPA, that H-abstraction was the dominant pathway via reaction with Cl radicals, constituting 79% of the reactive loss of BPA. Reactions with Cl_2_^•–^ are expected to be about an order of magnitude slower than those with Cl radicals but can also contribute to the degradation of BPA through H-atom abstraction. No evidence of such products was available from the mass spectra, which as described in the case of reaction between OH and BPA, may be due to a lower instrumental sensitivity for the oxidation products of BPA, concentrations that are below instrumental detection limits, or degradation and volatilization on the timescale of the experiment.

As shown in Figure 1, BPA continued to decay with increasing OH exposure levels above 4 × 10^11^ molecules s cm^–3^ in the binary-component BPA + NaCl aerosol, unlike for the pure-component BPA decay, which did not decay further with increasing OH exposure. As described in Sakamoto et al., if we considered OH reactions as occurring only on the surface of the aerosol, OH reactive uptake (i.e., decay rate of BPA in this study) would slow with increasing OH exposure. In contrast, no apparent limitations to OH reactive uptake exist when bulk reactions are considered. This highlights the potential importance of phase state or molecular diffusivity in the reactive uptake kinetics of OH and degradation of contaminant species in aerosol in general. NaCl is hygroscopic, and the high RH of the PAM-OFR (80%) was above its DRH. Here, the binary-component BPA + NaCl aerosol would hydrate, which would encourage mixing of reactive components in the aerosol phase. In contrast, BPA is hydrophobic and sparingly soluble in water, and thus no appreciable water uptake is expected in the pure-component BPA aerosol experiments even at this RH. To investigate this further, we performed theoretical calculations of the viscosity of pure-component BPA aerosol at RH = 80% and contrasted with the estimated viscosity of binary-component BPA + NaCl aerosol at RH = 80% (full details included in the SI).^53^ We considered two different conditions, one whereby the hygroscopicity parameter of BPA was set to κ = 0.01 (characteristic of sparingly water-soluble organic matter) and one whereby κ = 0.1 (often used for parameterizing the hygroscopicity of organic aerosol).^54^ In the case of κ = 0.1 for BPA, the viscosity of pure-component BPA aerosol at RH = 80% and a temperature of 295 K was a factor of two more viscous compared to the binary-component BPA + NaCl aerosol at the same temperature and RH. Assuming κ = 0.01 for BPA, pure-component BPA aerosol was nearly a factor of 10 more viscous compared to the binary-component BPA + NaCl aerosol. This indicates that the bulk diffusivity and mixing timescales of BPA, OH, and RCS were approximately 2 to 10 times faster in the presence of NaCl than in the pure-component BPA aerosol, which could explain the factor of four enhancement in the reactive uptake and degradation rates of BPA in the presence of NaCl. In addition to these viscosity calculations, we also applied the parameterization from Davies and Wilson, described in the BPA only section to the BPA + NaCl case and shown in Figure 1 (further details in the SI).^44^ In this case, the *k*_OH_ values for the linear and parameterized exponential fits were within error of each other, further indicating that in the presence of NaCl, the particle was well-mixed, which can lead to greater replenishment of unreacted BPA at the surface and thus greater fractional loss of BPA in the aerosol phase. In other mixed organic/inorganic aerosol systems, the phase state and RH are shown to regulate heterogeneous oxidation.^55^ This suggests further that uptake and reactions between OH and BPA were enhanced and more likely driven by both surface and bulk reactions in the binary-component BPA + NaCl aerosol with lower viscosity, whereas OH oxidation of BPA was limited to the surface in the more viscous pure-component BPA aerosol.

The Setschenow (salting-out) coefficient of BPA in NaCl has been measured as 0.174 M^–1^ .^56^ The polarity of the di-substituted phenyl rings suggests that BPA may have a propensity for both the bulk and the surface of the particles even with high ionic strength. Given the increased ionic strength of the particles with NaCl, however, some BPA is likely to be promoted to the particle surface. In addition to the greater mixing of BPA in the less viscous aqueous aerosol matrix containing NaCl, the effect of salting out could accelerate reactions between OH and BPA as reactions with OH are expected to occur within the near-surface layer of the particle (predicted reacto-diffusive length of OH is ≤1 nm).^19^ This effect by salting out has been observed in other aerosol systems, e.g., in the accelerated oxidation of sulfur by O_3_ in the presence of optically trapped sodium thiosulfate/sucrose/aqueous droplets.^57^ Furthermore, the larger surface area-to-volume ratio (SA/V) of BPA in the mixed BPA/NaCl aerosol compared to pure BPA could lead to greater reactive loss of BPA as observed in the heterogeneous oxidation of squalane by reaction with OH on squalene-coated ammonium sulfate particles compared to pure squalane particles.^46^

### Degradation of BPA in the Presence of NaCl and Photosensitizers by OH

Mimicking the complexity of SSA, we added to the mixture of BPA and NaCl two photosensitizing molecules as substitutes for the chromophoric dissolved organic matter found in SSA: 4-benzoylbenzoic acid (4-BBA) and more complex humic acid (HA). Both 4-BBA and HA have been employed in previous work as representative photosensitizing molecules but notably may lack some of the key photosensitizing properties and chemical features of real chromophoric material present at the ocean surface, which includes enriched levels of nitrogen.^9,58^ These experiments were performed in the absence and in the presence of NaCl to elucidate possible additive effects on the degradation of BPA by NaCl and the photosensitizers. The decay rates of BPA in the ternary-component aerosol composed of either photosensitizer were considerably higher than those in the binary-component BPA + NaCl aerosol. *k*_OH_ for the BPA + NaCl aerosol mixture was 2.091 (±0.098) × 10^–12^ cm^3^ molecule^–1^ s^–1^, while *k*_OH_ for the BPA + NaCl + 4-BBA aerosol mixture was 2.016 (±0.064) × 10^–12^ cm^3^ molecule^–1^ s^–1^, and *k*_OH_ for the BPA + NaCl+HA aerosol mixture was 2.062 (±0.069) × 10^–12^ cm^3^ molecule^–1^ s^–1^, which are all within error of each other. These correspond to uptake rates of 0.546 (±0.017) for BPA + NaCl+4-BBA and 0.580 (±0.019) for BPA + NaCl+HA. This is expected, as there is the impact of the lowered viscosity with the addition of NaCl, which can lead to increased photodegradation by better mixing of the photosensitizer molecules, allowing more photosensitizer molecules to be activated by light at the surface of the particle. *k*_OH_ = 1.226 (±0.047) × 10^–12^ cm^3^ molecule^–1^ s^–1^ in the presence of 4-BBA, and *k*_OH_ = 0.791 (±0.008) × 10^–12^ cm^3^ molecule^–1^ s^–1^ in the presence of HA. This corresponds to γ = 0.313 (±0.012) and γ = 0.210 (±0.002), respectively, which was an enhancement of up to 49% in γ for the BPA mixture with 4-BBA. Interestingly, the photosensitizers behave quite differently without NaCl present. Analogous to our findings, Trueblood et al. reported that HA was a less efficient photosensitizer than 4-BBA with respect to the degradation of nonanoic acid thin films, although a mechanism was not provided. However, the larger molecular weight and predicted viscosity of HA in comparison with 4-BBA and BPA could lead to morphology changes at the high RH in this study, including phase separation between organic components with different solubilities, in analogy to the liquid–liquid phase separation as observed for mixed aqueous organic–inorganic aerosol.^59^ If phase separation occurred, we speculate that the slower degradation of BPA in the aerosol mixture with HA was due to less interaction of reactive species formed by HA under light exposure.

### Degradation of BPA in the Presence of 254 nm and 369 nm Light

**Figure 4:**
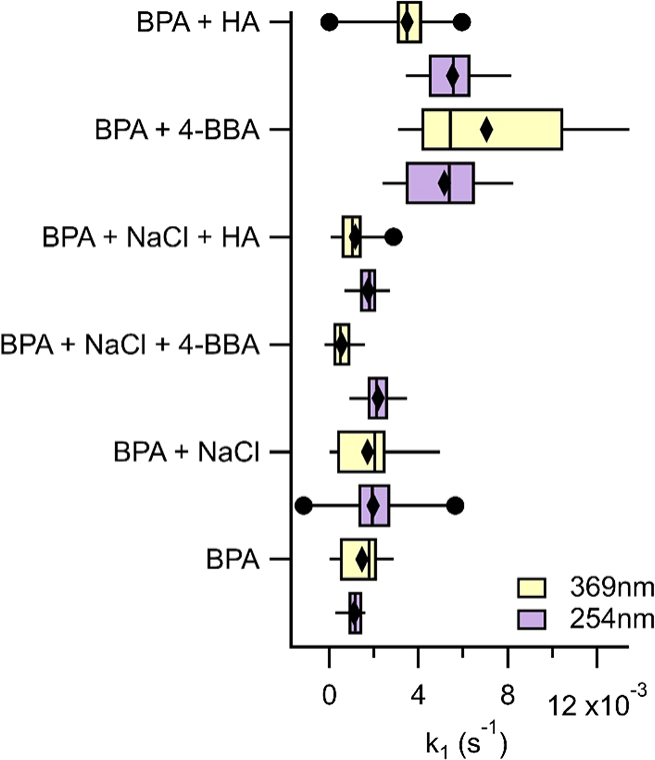
First-order loss rates of BPA for the different aerosol systems following exposure to 369 nm light (yellow boxes) and 254 nm light (purple boxes). Median values are shown by the horizontal line, the 25th and 75th percentiles are bounded by the box, the 5th and 95th percentiles are shown by the whiskers, the black diamonds represent the mean, and the black dots represent the outliers. *N* = 15 for each aerosol system.

In a separate set of experiments, BPA was exposed to either 254 nm light or 369 nm light inside the PAM-OFR in the absence of OH and ozone but with the same RH and flow rates as in all experiments. In the 254 nm experiments, the irradiance was adjusted systematically from 24 to 171 W/m^2^, which corresponds to the same levels in the OH experiments. In the 369 nm experiments, the irradiance was adjusted from 30 to 267 W/m^2^. First-order loss rate constants, *k*_254_ and *k*_369_, were calculated from the slope of the natural logarithm of the fractional decay of BPA (before and after irradiance) as a function of the residence time (123 s) in the PAM-OFR. The results for the different aerosol systems are displayed in Figure 4. *k*_369_ for the pure-component BPA was 1.457 (±1.027) × 10^–3^ s^–1^ and *k*_254_ was 1.153 (±0.398) × 10^–3^ s^–1^. In the binary-component BPA + NaCl aerosol mixture, mean *k*_369_ = 1.711 (±1.418) × 10^–3^ s^–1^ and *k*_254_ = 1.928 (±1.626) × 10^–3^ s^–1^, which were not statistically different from the first-order decay rates of pure-component BPA aerosol upon irradiation. Irradiation of the ternary-component BPA + NaCl + photosensitizer aerosol by 369 nm light led to a slight but insignificant decrease in the degradation of BPA compared to the binary-component BPA + NaCl aerosol. Here, *k*_369_ = 0.545 (±0.512) × 10^–3^ s^–1^ and *k*_254_ = 2.105 (±0.772) × 10^–3^ s^–1^ for the BPA + NaCl+4-BBA aerosol mixture and *k*_369_ = 1.168 (±0.843) × 10^–3^ s^–1^ and *k*_254 =_ 1.801 (±0.586) × 10^–3^ s^–1^ for the BPA + NaCl+HA aerosol mixture. In contrast, in the absence of NaCl, the addition of the photosensitizers to BPA led to a significant enhancement in the degradation of BPA following exposure to 369 nm light, increasing to *k*_369_ = 7.050 (±3.821) × 10^–3^ s^–1^ and *k*_254 =_ 5.381 (±1.797) × 10^–3^ s^–1^ for the binary-component BPA + 4-BBA and *k*_369_ = 3.478 (±1.375) × 10^–3^ s^–1^ and *k*_254_ = 5.577 (±1.373) × 10^–3^ s^–1^ for the binary-component BPA + HA aerosol mixtures. Zhan et al. found similarly that the rate of BPA degradation in bulk aqueous solutions under solar-simulated light was insignificant with BPA alone but increased in the presence of humic acids.^28^

It is possible that BPA degradation in the ternary-component aerosol mixtures was suppressed due to quenching of the excited triplet state of the photosensitizer by halide ions. As discussed in Tinel et al. and shown in eq 6, the excited triplet state of the photosensitizer, imidazole-2-carboxaldehyde (IC), which has been shown in the other work by Tinel et al. to exhibit similar photosensitizing capabilities as 4-BBA, gets quenched in the presence of halides:^60,61^
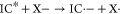
6

In the absence of NaCl, the photosensitizers can access the excited triplet state and upon energy transfer can degrade neighboring organic molecules via formation of reactive oxygen species. Due to its lower activation energy, OH reactions are expected to dominate compared to Cl radicals and other reactive chlorine species such as ClO radicals. Thus, although the corresponding halide radical is formed upon quenching, photosensitized production of OH and other reactive oxygen species gets suppressed, leading to slower rates of BPA degradation than what was observed in the absence of NaCl. The greater loss rates measured for BPA in the binary-component BPA + photosensitizer aerosol mixtures following exposure to 254 nm and 369 nm radiation are attributed to photosensitized degradation processes. Singlet oxygen can be produced in the presence of UV/VIS radiation due to an electronic energy transfer between the excited triplet state of the photosensitizer and the ground triplet state of molecular oxygen, which could readily react with BPA.^23,62^ Alternatively, direct interactions between the triplet state of the photosensitizer and BPA can promote the formation of radicals such as HO_2_, accelerating the loss of BPA when irradiated.^63^ Notably, there is little difference between the first-order loss rates of BPA mixed with the photosensitizers when exposed to 254 nm light compared to irradiation at 369 nm, which is indicative that these photosensitized processes do not require absorption of highly energetic light to proceed, and could proceed under more relevant daylight conditions in the atmosphere. Using the Atmospheric Chemistry Observations & Modeling tool from the National Center for Atmospheric Research, it is estimated that during an average summertime afternoon, San Diego, CA would receive roughly 2.02 × 10^13^ photons s^–1^ cm^–2^ of 369 nm light.^64^ While the lowest light exposure in the 369 nm experiments was still an order of magnitude greater than that predicted by the model, light exposures are expected to increase with altitude to ∼1 × 10^14^ photons s^–1^ cm^–2^ at 369 nm and 6 km in the atmosphere. Additionally, integrated light exposure across all UV and VIS wavelengths of the solar spectrum could lead to even greater photosensitized degradation rates of BPA than what was reported here at only 369 nm. Furthermore, longitude and solar zenith angle (or time of day) will affect the intensity and wavelength of light absorbed by the aerosol.

## Conclusions

In this study, we demonstrated that OH-initiated heterogeneous reactivity and photodegradation kinetics of aerosol-phase BPA, representative of the toxic additives in airborne micro- and nano-plastics, are greatly impacted by the presence of sea salt (NaCl) and photosensitizing species. The heterogeneous and photosensitized processes that promote the decay of BPA depend on the particle viscosity or phase state. Aerosol mixtures composed of BPA and NaCl, the least viscous phase states, exhibited the greatest loss of BPA following exposure to OH due to greater diffusive mixing of reactive species in the particle bulk. Calculated uptake coefficients based on the decay of BPA were a factor of four greater in the presence of NaCl compared to pure-component BPA aerosol, and bulk diffusion mixing timescales of reactants in the particle phase were shown to be a factor of 2 to 10 faster in the presence of NaCl. The addition of a photosensitizer to BPA promoted the loss of BPA following light exposure to 369 nm radiation in the absence of OH. No significant enhancements in the reactive losses of BPA by OH or light exposure were observed when a photosensitizer was added to the BPA + NaCl aerosol mixtures. These differences were attributed to surface-enhanced photosensitized reactions in the case of the more viscous BPA + photosensitizer aerosol and quenching of the excited triplet state of the photosensitizer in the more liquid-like aqueous-phase NaCl aerosol.

In the context of SSA in the real atmosphere, particularly in the marine boundary layer where the RH is ∼85% or greater, photosensitized reactions between chromophores emitted in SSA and plastic contaminant molecules such as BPA may be limited due to quenching of the triplet state by halide ions, whereas heterogeneous oxidation via OH could promote the formation of reactive halogen radicals. The more liquid-like phase states of SSA in the marine boundary layer^18^ could increase the probability of reaction with BPA as OH, BPA, and RCS diffuse more readily in the particle bulk in the presence of aqueous sea salt, promoting the degradation of BPA. As contaminated SSA becomes lofted over land and mixed in the vertical to higher altitudes above the marine boundary layer to regions of lower RH and exposure to more intense solar radiation, the particles dry and BPA and chromophoric organic molecules get salted out and promoted to the particle surface wherein photosensitized reactions may become more prevalent.

**Figure 5:**
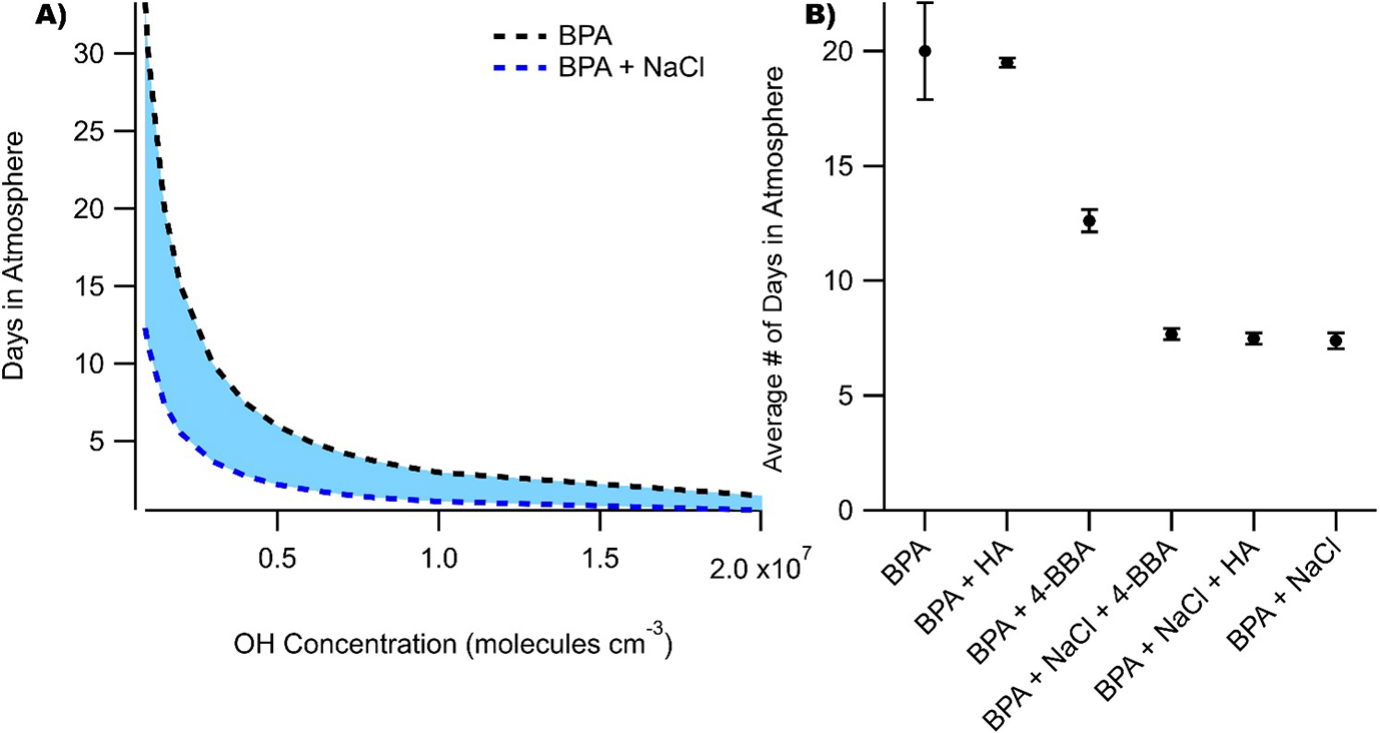
(A) Chemical lifetimes of BPA with respect to heterogeneous OH oxidation as a function of OH concentration. (B) Averaged chemical lifetimes of BPA in the different aerosol mixtures based on [OH] = 1.5 × 10^6^ molecules cm^–3^.

The e-folding lifetimes (τ) of BPA following exposure to OH in the different aerosol mixtures studied here are shown in Figure 5A and were calculated from eq 7:
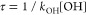
7

The e-folding lifetime is equivalent to the average time that BPA would remain chemically viable in aerosol and calculated assuming [OH] = 1.5 × 10^6^ molecules cm^–3^. BPA alone is expected to decay due to heterogeneous reaction with OH with a τ equivalent to about three weeks in the atmosphere, which is longer than the physical lifetime of the aerosol of around a week due to wet deposition. In the presence of NaCl, the lifetime of BPA is reduced by more than half to 7.4 days, competitive with wet depositional lifetimes. In drier areas like the Pacific Southwest where rain events are infrequent, heterogeneous and photo-initiated oxidation processes could thus serve as the major sinks for airborne particulate BPA. In the absence of NaCl but presence of a photosensitizer, the chemical lifetime of BPA is reduced with respect to pure-component BPA to 12.6 days in the presence of 4-BBA and 19.5 days in the presence of HA. These timescales are important as they suggest that BPA and other additives in plastics that become airborne can remain in appreciable amounts in the aerosol phase for up to a week following their emission.

Future work could aim to increase the complexity of the aerosol to understand how BPA degrades in real SSA matrices, e.g., by generating SSA from bubble-bursting in real seawater employing a marine aerosol reference tank.^65^ The composition of the chromophores in the dissolved organic matter in real SSA can differ from 4-BBA and HA studied here, potentially leading to different photosensitized degradation rates.^9^ In addition, photosensitized reactions and degradation rates are known to differ depending on whether the organic matter is in its anionic or neutral (zwitterionic) forms and shown to increase with increasing pH.^66^ In the work of Angle et al., the pH of SSA was shown to vary with particle size and differ from the pH of seawater at the ocean–air interface.^67^ Although the pH of the atomizer solutions (neutral to slightly acidic) indicated that BPA was in its molecular form and 4-BBA was in its carboxylate form, the pH of the aerosol could be important in dictating the photosensitized kinetics, requiring further study beyond the scope of this work. Overall, this study provides a basis for understanding the different heterogeneous and photochemical degradation pathways of a key atmospheric plastic additive present in microplastics, which is an emerging concern for air quality in coastal marine environments.

## Supporting Information Available

BPA signal correction method, radial reaction depth modeling for BPA only and BPA + NaCl, estimated glass transition temperature and viscosity calculations for pure component and salt-containing BPA aerosol, BPA full time series with corrections (Figure S1), particle mass change with oxidation (Figure S2), evidence of chloride depletion (Figure S3) (PDF)
