# Supplementary material for: Heterogeneous and Photosensitized Oxidative Degradation Kinetics of the Plastic Additive Bisphenol-A in Sea Spray Aerosol Mimics
Source: J Phys Chem A. 2023 May 18;127(21):4724–33. doi: 10.1021/acs.jpca.3c00127 (PMC10240490; doi:10.1021/acs.jpca.3c00127)
Supplement: Supplementary file 1 — jp3c00127_si_001.pdf [file jp3c00127_si_001.pdf]

# Supporting Information:

Heterogeneous and Photosensitized Oxidative Degradation Kinetics of the Plastic Additive Bisphenol-A in Sea Spray Aerosol Mimics

*Samantha M. Kruse & Jonathan H. Slade**

Department of Chemistry and Biochemistry, University of California, San Diego, La Jolla, California 92093, United States

*Corresponding author email: jhslade@ucsd.edu

## Bisphenol-A (BPA) Signal Correction Method

We applied a single-point standardization of the EESI-TOF during the experiment to monitor potential drifts in sensitivity. Here, atomized BPA aerosol periodically bypassed (byp) the PAM-OFR. The adjusted ($I_{adj}$) BPA signal was calculated using formula Eq. S1:

$$I_{adj} = I_N - \left(I_N\left(\frac{I_{byp,\,2}}{I_{byp,\,N}}\right)\right) \text{ (Eq. S1)}$$

Here, $I_N$ represents the BPA signal (ions $s^{-1}$) while the aerosol is flowing through the PAM-OFR during OH exposure (red shaded regions in Fig. S1), $I_{byp,2}$ is the signal of the second single point standard (the second grey shaded region in Fig. S1), and $I_{byp,N}$ is the signal of the single point standard that correlates to $I_N$. The first grey shaded region in Fig. S1 is not utilized for correction because everything is normalized to the signal of aerosol flow through the PAM-OFR with no OH exposure (region in Fig. S1 with no shading) and is only used to ensure proper instrument function after the filter blank (green shaded region in Fig. S1) before starting the experiment.

## Radial Reaction Depth Modeling for BPA Only and BPA+NaCl

To investigate the nonlinearity of the BPA only system, we chose to incorporate the parameterization from Davies and Wilson 2015.[1] In this model, we used the following equation to determine the effective second order rate constant, $k_{OH}$, radial reaction depth, $L$, and the radial probing depth, $d$:

$$\ln\left(\frac{[BPA]}{[BPA_0]}\right) = \ln\left(\frac{D^3-(D-2L)^3}{D^3-(D-2d)^3} exp\left(-k_{OH}x\right) + \frac{(D-2L)^3-(D-2d)^3}{D^3-(D-2d)^3}\right)$$

In this equation, $D$ is the diameter of the particle, and [BPA]/[$BPA_0$] is the concentration as determined from the EESI-MS. Initially, $k_{OH}$ was allowed to vary, and in the case of BPA and NaCl, this $k_{OH}$ value was within that of the linear fit, indicating that the particles were well-mixed. However, in the case of BPA only, the values varied significantly, so the data was fit again, allowing $k$, $L$, and $d$ to vary. In the case of EESI-MS, $d$ is not defined, as it is dependent on the RH and solubility of the sample molecules in the reagent spray, so it was also determined by the fitting procedure. In this fitting, $L$ was determined to be 2.5 (± 1) nm, $k_{OH}$ was determined to be 5.93 (± 1.23) × $10^{-12}$ $cm^3$ $molecule^{-1}$ $s^{-1}$, and $d$ was determined to be 9 (± 5) nm. After these parameters were determined, the new gamma was calculated using the following eq from Davies and Wilson 2015:

$$\gamma = \frac{2*\rho_{aq}*m_f*N_A}{3*c*M_{org}*k_{OH}} * \frac{D^3-(D-2L)^3}{D^2}$$

Where $m_f$ is the mass fraction of BPA, $\rho_{aq}$ is the density of BPA, $N_A$ is Avogadro's number, c is the mean velocity of OH, $M_{org}$ is the molar mass of BPA, and $k_{OH}$ is the effective second order rate constant determined in the fitting procedure. This gives us gamma of 0.268 (± 0.056), which is within error of the gamma calculated based off the linear fit, 0.247 (± 0.026).

## Estimated Glass Transition Temperature and Viscosity Calculations for Pure Component and Salt-Containing BPA Aerosol

The methods shown here closely follow those from Tumminello et al 2021.[2] Viscosity of the BPA aerosol was estimated from the aerosol's glass transition temperature ($T_g$), defined as the approximate range of temperatures over which a transition between semi-solid to amorphous solid (glassy) states occur.[3,4] Here, we estimate the dry $T_g$ of BPA ($T_{g,org}$) applying the formulations described in DeRieux et al.[5] $T_{g,org}$ was calculated using Eq. S2, where M and O:C are the molar mass and O:C of BPA with M=227 g $mol^{-1}$, O:C=0.13, and A, B, C, D, and E are best fit parameters.[5]

$$T_{g,\,org} = A + BM + CM^2 + D(O{:}C) + EM(O{:}C) \text{ (Eq. S2)}$$

To account for the impact of aerosol liquid water content on the $T_{g,org}$ of BPA, we calculated the volume of liquid water associated with BPA ($V_{w,org}$) in equilibrium with the relative humidity (assuming the water activity, $a_w$, is equal to relative humidity) applying Eq. S3.[6]

$$V_{w,org} = \frac{a_w}{1 - a_w} V_{org} \varepsilon_{org} \kappa_{org} \text{ (Eq. S3)}$$

The volume of the organic component in SSA ($V_{org}$) was determined based on the mean surface-area weighted diameter of 100 nm and the organic volume fraction ($\varepsilon_{org}$) from Eq. S4:

$$\varepsilon_{org} = \frac{f_{org}}{\left(f_{org} + (1 - f_{org})\left(\frac{\rho_{org}}{\rho_{org}}\right)\right)} \text{ (Eq. S4)}$$

Here, $f_{org}$ is the mass fraction of BPA, and $\rho_{org}$ and $\rho_{inorg}$ refer to the densities of the organic material (1.2 g $cm^{-3}$) and inorganic material (2.2 g $cm^{-3}$),[7] respectively. The volume mixing rule[6] was applied to solve for $\kappa_{org}$ based two $\kappa$ assumptions of 0.1 and 0.01 and assuming $\kappa_{inorg}$=1.51 for NaCl.[8] Note that there are certain assumptions in the calculated $\kappa_{org}$ applying the volume mixing rule, including (1) that the aerosol population is considered homogeneous and contains both salt and organic material as an internal mixture, (2) the hygroscopicity parameter of the inorganic component is known, and (3) aerosol water uptake behaves as a linear combination of the organic and inorganic components. $V_{w,org}$ was converted to mass of water ($m_{w,org}$) from the density of water of 1.0 g $cm^{-3}$. The Gordon-Taylor equation (Eq. S5) was applied to calculate the

average $T_g$ of the organic-water mixture, $T_{g,org,w}$, where $f_{ALW}= m_{w,org}/(m_{org}+m_{w,org})$ and $T_{g,w}$ is the glass transition of pure water (136 K).[5]

$$T_{g,org,w} = \frac{T_{g,w}(f_{ALW}) + \frac{1}{k_{GT}} f_{org} T_{g,org}}{(f_{ALW}) + \frac{1}{k_{GT}} f_{org}} \text{ (Eq. S5)}$$

Finally, $T_{g,org,w}$ values were converted to viscosity, $\eta_{org}$ (Pa·s) utilizing Eqs. S6 and S7, where z, the fragility parameter was set to 12, a value used for organic compounds in previous studies.[4,5]

$$T_0 = \frac{39.17 T_{g,org,w}}{z + 39.17} \text{ (Eq. S6)}$$

$$\log \eta = -5 + 0.434 \frac{T_0 z}{T - T_0} \text{ (Eq. S7)}$$

The viscosities of BPA and NaCl were calculated separately and then combined to estimate the viscosity of the mixture, which assumes the organic and inorganic components are homogeneous and internally-mixed, so we caution the following calculations are only appropriate for homogeneous internally-mixed organic/inorganic salt particles. To determine the viscosity of the aqueous salt solution, we first calculated the volume fraction of the inorganic portion (assuming an equal atomization efficiency of 0.5 for NaCl and 0.5 for BPA) ($\varepsilon_{inorg}$) as $\varepsilon_{inorg} = 1 - \varepsilon_{org}$, where $\varepsilon_{org}$ was derived from Eq. S4. We then estimated the aerosol liquid water derived from the inorganic components in analogy to that for the organic components using Eq. S4 and applying a $\kappa_{inorg}$=1.51 for NaCl.[8] The viscosity of the aqueous salt solution, $\eta_{inorg}$, was calculated adapting the formulations described in Goldsack and Franchetto, 1976.[9] In its simplest form, the $\eta_{inorg}$ can be calculated using Eq. S8:

$$\eta_{inorg} = \frac{\eta_w e^{X_c E}}{1 + X_c V} \text{ (Eq. S8)}$$

Here, $\eta_w$ is the viscosity of water at 25°C, 0.8904 cP, $X_c$ is the mole fraction of cations in solution, defined as $X_c=m_c/(55.51+2m)$, which applies to an aqueous solution on the molal scale given there are 55.51 moles of water per kg of water and 2m moles of salt (anion plus cation) for an m molal solution of a 1:1 salt, which we apply here for NaCl. E is the free energy based on the relationship, $E=(\Delta G_c^* + \Delta G_a^* - 2\Delta G_1^*$ 116 )/RT, determined from a data table (Table 3 in Goldsack and Franchetto, 1976)[9] for NaCl. V is the molar volume of the hole in the liquid

created by the cations and anions moving in the liquid, also determined from the same data table. Assuming the organic components and salt are well mixed in the aerosol, the component viscosities of each were combined to calculate the viscosity of the BPA/NaCl aerosol based on the following classic mixing rules for liquids[10]:

$\ln\left(\eta_{mix}\right) = \sum_{i=1}^{N} x_i \ln\left(\eta_i\right)$ (Eq. S9)

Here, $x_i$ is the mole fraction and $\eta_i$ is the viscosity of the component, i=organic or inorganic salt.

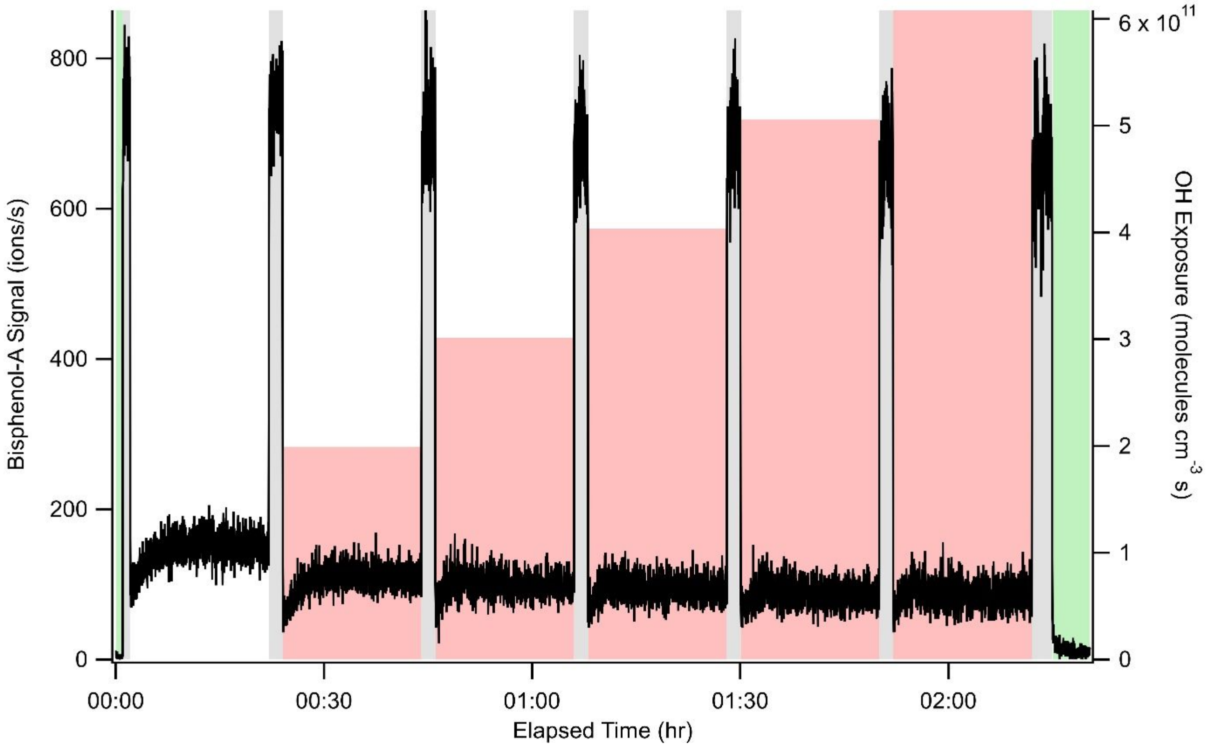


**Figure S1.** Full time series of BPA signal for one experiment. The green shaded regions indicate filter blanks, the grey shaded regions indicate the internal standards, and the red shaded regions indicate the OH exposures, corresponding to the right y-axis.

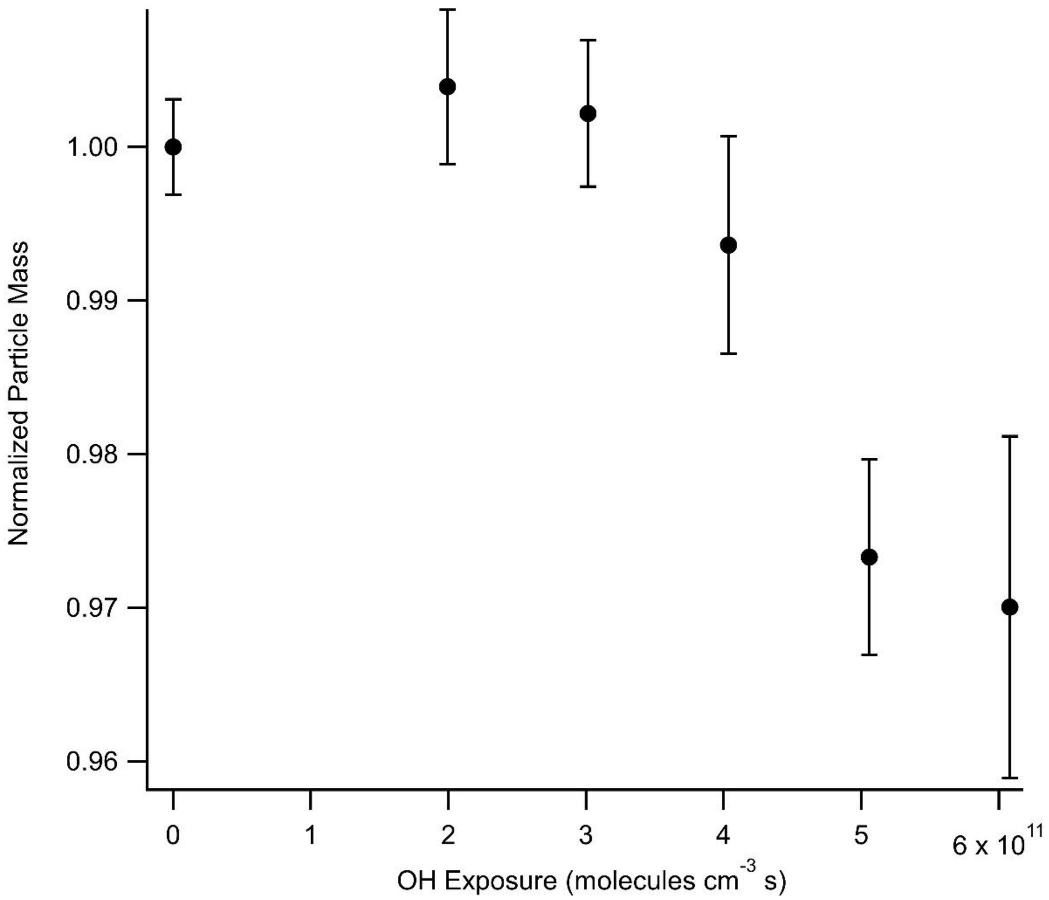


**Figure S2.** Normalized pure-component BPA aerosol mass concentrations as a function of OH exposure in the PAM-OFR.

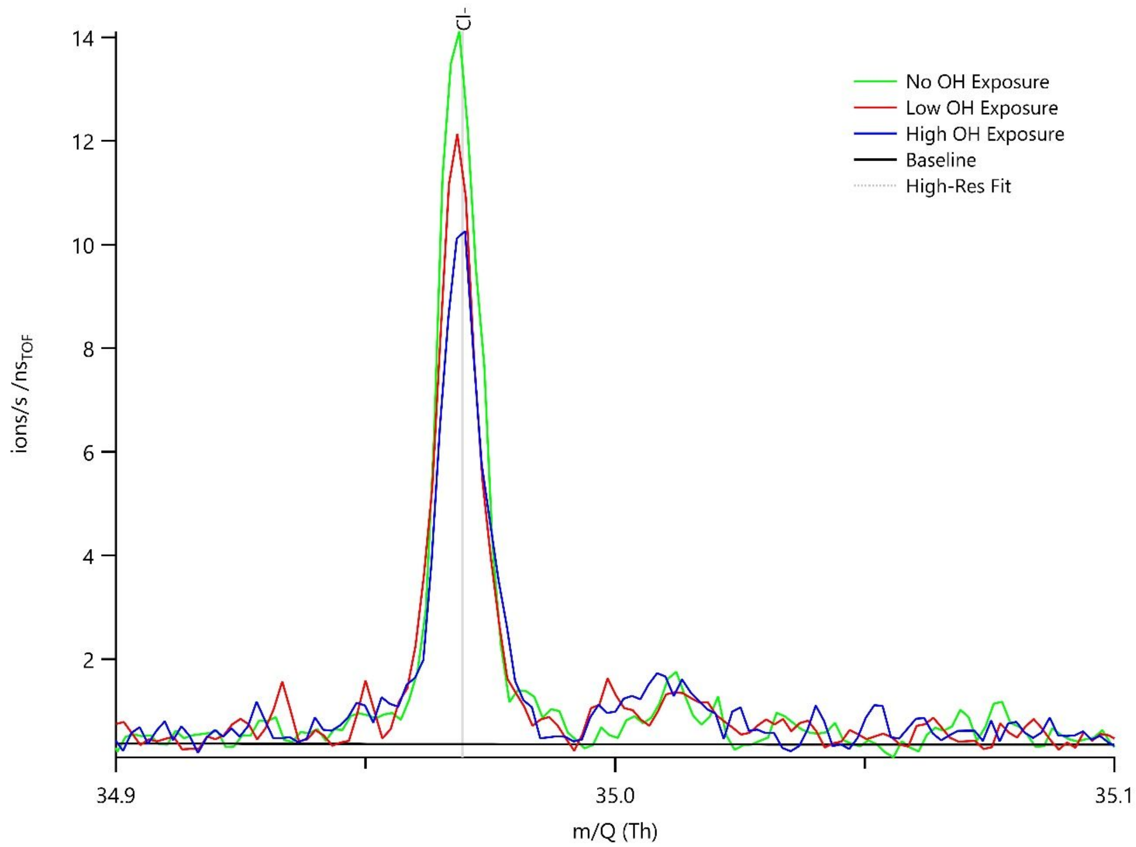


**Figure S3.** Chloride signal (m/z= 34.969401 Th) at no OH exposure (green), the lowest OH exposure (red), and the highest OH exposure (blue). Black represents the fitted spectrum baseline, and the light grey line represents the high-resolution fit for the no OH exposure signal.

## References


(1) Davies, J. F.; Wilson, K. R. Nanoscale Interfacial Gradients Formed by the Reactive Uptake of OH Radicals onto Viscous Aerosol Surfaces. *Chem. Sci.* **2015**, *6* (12), 7020–7027.

(2) Tumminello, P. R.; James, R. C.; Kruse, S.; Kawasaki, A.; Cooper, A.; Guadalupe-Diaz, I.; Zepeda, K. L.; Crocker, D. R.; Mayer, K. J.; Sauer, J. S. et al. Evolution of Sea Spray Aerosol Particle Phase State Across a Phytoplankton Bloom. *ACS Earth Space Chem.* **2021**, *5* (11), 2995–3007.

(3) Koop, T.; Bookhold, J.; Shiraiwa, M.; Pöschl, U. Glass Transition and Phase State of Organic Compounds: Dependency on Molecular Properties and Implications for Secondary Organic Aerosols in the Atmosphere. *Phys. Chem. Chem. Phys.* **2011**, *13* (43), 19238–19255.

(4) Shiraiwa, M.; Li, Y.; Tsimpidi, A. P.; Karydis, V. A.; Berkemeier, T.; Pandis, S. N.; Lelieveld, J.; Koop, T.; Pöschl, U. Global Distribution of Particle Phase State in Atmospheric Secondary Organic Aerosols. *Nat. Commun.* **2017**, *8* (1), 15002.

(5) DeRieux, W.-S. W.; Li, Y.; Lin, P.; Laskin, J.; Laskin, A.; Bertram, A. K.; Nizkorodov, S. A.; Shiraiwa, M. Predicting the Glass Transition Temperature and Viscosity of Secondary Organic Material Using Molecular Composition. *Atmos. Chem. Phys.* **2018**, *18* (9), 6331–6351.

(6) Petters, M. D.; Kreidenweis, S. M. A Single Parameter Representation of Hygroscopic Growth and Cloud Condensation Nucleus Activity. *Atmos. Chem. Phys.* **2007**, *7* (8), 1961–1971.

(7) Collins, D. B.; Zhao, D. F.; Ruppel, M. J.; Laskina, O.; Grandquist, J. R.; Modini, R. L.; Stokes, M. D.; Russell, L. M.; Bertram, T. H.; Grassian, V. H. et al. Direct Aerosol Chemical Composition Measurements to Evaluate the Physicochemical Differences between Controlled Sea Spray Aerosol Generation Schemes. *Atmos. Meas. Tech. Discuss.* **2014**, *7* (7), 6457–6499.

(8) Topping, D.; Barley, M.; Bane, M. K.; Higham, N.; Aumont, B.; Dingle, N.; McFiggans, G. UManSysProp v1.0: An Online and Open-Source Facility for Molecular Property Prediction and Atmospheric Aerosol Calculations. *Geosci. Model Dev.* **2016**, *9* (2), 899–914.

(9) Goldsack, D. E.; Franchetto, R. The Viscosity of Concentrated Electrolyte Solutions. I. Concentration Dependence at Fixed Temperature. *Can. J. Chem.* **1977**, *55* (6), 1062–1072.

(10) Grunberg, L.; Nissan, A. H. Mixture Law for Viscosity. *Nature* **1949**, *164* (4175), 799.
